# Error, Power, and Blind Sentinels: The Statistics of Seagrass Monitoring

**DOI:** 10.1371/journal.pone.0138378

**Published:** 2015-09-14

**Authors:** Stewart T. Schultz, Claudia Kruschel, Tatjana Bakran-Petricioli, Donat Petricioli

**Affiliations:** 1 Department of Ecology, Agriculture, and Aquaculture, University of Zadar, M. Pavlinovica bb, 23000 Zadar, Croatia; 2 Department of Biology, Faculty of Science, University of Zagreb, 10000 Zagreb, Croatia; 3 D.I.I.V. Ltd, 23281 Sali, Croatia; Università della Calabria, ITALY

## Abstract

We derive statistical properties of standard methods for monitoring of habitat cover worldwide, and criticize them in the context of mandated seagrass monitoring programs, as exemplified by *Posidonia oceanica* in the Mediterranean Sea. We report the novel result that cartographic methods with non-trivial classification errors are generally incapable of reliably detecting habitat cover losses less than about 30 to 50%, and the field labor required to increase their precision can be orders of magnitude higher than that required to estimate habitat loss directly in a field campaign. We derive a universal utility threshold of classification error in habitat maps that represents the minimum habitat map accuracy above which direct methods are superior. Widespread government reliance on blind-sentinel methods for monitoring seafloor can obscure the gradual and currently ongoing losses of benthic resources until the time has long passed for meaningful management intervention. We find two classes of methods with very high statistical power for detecting small habitat cover losses: 1) fixed-plot direct methods, which are over 100 times as efficient as direct random-plot methods in a variable habitat mosaic; and 2) remote methods with very low classification error such as geospatial underwater videography, which is an emerging, low-cost, non-destructive method for documenting small changes at millimeter visual resolution. General adoption of these methods and their further development will require a fundamental cultural change in conservation and management bodies towards the recognition and promotion of requirements of minimal statistical power and precision in the development of international goals for monitoring these valuable resources and the ecological services they provide.

## 1 Introduction

Conservation monitoring, the regular observation of a valuable environmental resource, is the cornerstone of natural resource management programs worldwide [1]. Its purpose is to identify where and when that resource is in decline, so that prompt recovery and protection actions can be taken. It functions metaphorically as a sentinel, whose purpose is to provide the first alert to the presence of threats, as signalled by negative changes in the resource [2]. If monitoring is not capable of reliably recognizing decline as it first occurs, then it is a failure as a sentinel, and it fails the management program that depends on it [3]. Nevertheless, to outside observers the program may, for a time, appear both responsible and successful, because it is built on a formally approved monitoring protocol, and at least initially, that protocol does not show any significant loss in the resource it is charged to watch.

The two major obstacles to successful monitoring are 1) natural variation in the resource, and 2) inherent methodological error or uncertainty [4]. Both introduce random or systematic noise into any measurement of loss. If the noise overwhelms the signal, then the method cannot distinguish signal from noise. If it cannot distinguish signal from noise, then the method is no better than a coin toss, and is in practical terms a blind sentinel. If a management program relies on such a method for surveillance of a threatened resource, then this reliance effectively guarantees the loss of the resource. Such losses have been documented repeatedly on four continents [5–7]. For this reason, such methods are often considered worse than no monitoring at all [5, 8], since investing alternatively in other kinds of knowledge, such as patterns of human disturbance known to cause habitat degradation, will likely provide more predictive power than a coin toss [9].

Several recent reviews of environmental monitoring programs worldwide have indicated that very few such programs are statistically powerful enough to detect losses in the resources for which they are responsible. For example, in Australia “the record on monitoring is appalling for all government agencies involved in forest management” [7], and worldwide “millions of dollars are currently being wasted on monitoring programmes that have no realistic chance of detecting changes in the variables of interest” [5]. These problems have created an ongoing “crisis of credibility in the value and relevance of the entire monitoring process” [6] because, ironically, the very measures created to expose the decline of valuable resources have instead obscured their decline, ensuring that those at risk will continue to decline undetected until large and likely irreversible losses occur [1, 5–7]. In practical terms, monitoring programs in many regions, both terrestrial and aquatic, are functioning as blind sentinels, unable to clearly perceive losses until the time has long passed for any meaningful conservation intervention. The main reason for this dysfunction is that conservation policy and management bodies, and the non-governmental organizations (NGOs) under contract with them, often do not have the pertinent scientific and technical expertise, and because they are not a part of the academic community, their study designs are not subject to peer review and they are generally not under any formal obligation to follow the advice of scientists [5]. They are a subset of the economy that is de facto scientific, but not beholden to any formal scientific body.

Scientists have known for decades that the commonly used methods worldwide for monitoring seagrass cover and density have unacceptably low statistical power, and therefore “can only detect a reliable tendency towards seagrass loss when the seagrass meadows monitored have already experienced substantial damage” ([10], p. 201). In the case of slow-growing species such as *Posidonia*, whose horizontal rhizomes have not been observed in nature to grow more than about 6 cm in one year, moderate loss beyond a local scale is catastrophic and unlikely to be reversed in the lifetime of managers [11, 12]. Such losses are costly; *Posidonia oceanica* and similar seagrasses are estimated to provide 1.72 million euros per year per hectare in ecosystem services (sediment accretion, erosion control, habitat, food web energy input, water purification; [13]). Duarte [10] pointed out that if we are serious about safeguarding this value, then the first management priority should be the development and use of monitoring methods with statistical power sufficient to detect a loss of 10% ([10], p. 202). This should probably be considered a bare minimum, with a target of 2–5% preferable, since an annual loss of 10% in a slow-growing species such as *Posidonia* would mean permanent irreversible loss in just a few years; a 10% loss of *Posidonia* translates to 172.000 euros lost per hectare per year. The ability to perceive a small loss is crucial for any early warning system.

However, the development of such methods has not been a priority in many management or regulatory bodies before or since these critical reviews. This is demonstrated by the absence of any requirement regarding minimum sample size, minimum power, minimum precision, or minimum detectable difference in formal published guidelines and legal requirements of government monitoring protocols currently in force internationally (Europe: [14–19]; USA: [20–23] Australia: [24–26]). As an example, the latest formal EU document that presents the requirements for an approved monitoring protocol for the Natura2000 system in Croatia under the European Commission (EC) Habitats Directive [14] does not even address the subject of statistical power or minimum detectable difference, let alone establish minimum requirements, though it would be easy to do so.

This fundamental statistical gap leaves open the possibility that a monitoring protocol can be proposed in many jurisdictions worldwide that strictly satisfies every formal requirement and follows every guideline, is approved by the pertinent state and national agencies responsible for nature protection, but nevertheless is a blind sentinel, perhaps with good intentions, but still incapable of detecting real losses until it is too late. Lindenmayer and Gibbons [27] refer to this as the “it’s the thought that counts” approach, which is common worldwide: a government power mandates regular sentinel monitoring, and mandates intervention based on monitoring results, but neglects to mandate that the approved monitoring protocol actually works.

Although excellent reviews of seagrass monitoring descriptors and methods are available [28–31], there is as yet no critical comparison of their statistical power. That monitoring of *Posidonia* has failed at the regional scale is suggested by a recent review concluding that *Posidonia* cover may have declined 13% to 50% throughout the Mediterranean from 1842 to 2009, and shoot density may have declined 50% during just the last 20 years [32], in spite of regular, mandated monitoring in several Mediterranean countries. Given these facts, can monitoring still serve a purpose as a reliable sentinel?

Marba and colleagues [30] make a call for action: “we strongly encourage the evaluation of seagrass indicator-pressure responses and quantification of the uncertainty of classification associated to the indicator in order to identify the most effective seagrass indicators for assessing ecological quality of coastal and transitional water bodies.” This paper is in part a response to this call for action. Our purpose is to consider the statistical power in detecting a 10% loss for common seagrass monitoring methods, as applied to *Posidonia* in the Mediterranean Sea. The basic questions we ask are 1) what effect does spatial variability in cover and density have on statistical power and minimum sample size for detecting a 10% loss? 2) what is the statistical benefit of fixed-plot direct methods for measuring a 10% loss in habitat cover? 3) what are the statistical consequences of habitat classification error present in remote methods that use acoustic or visual maps to assess loss in habitat cover? and finally 4) what methods are currently available that do satisfy the 10% criterion or better, and hence can be considered reliable sentinels?

## 2 Methods and Results

### 2.1 Monitoring methods

We assume a monitoring decision tree in which there are three major statistical decision nodes, namely 1) direct versus indirect (remote) sampling methods, 2) fixed- versus random-plot methods, and 3) presence versus absence of habitat classification error. Prior to these nodes is a non-statistical decision between destructive and non-destructive methods; here we consider only non-destructive.

Direct methods are those in which personnel carry out all observations in situ, which has the benefit of direct observation, but a high labor cost, especially for subtidal habitats that require trained SCUBA divers for all in situ work. Indirect or remote methods as defined here are those in which the sensor or personnel are above water. These have the benefit of rapid image capture of large areas, but the disadvantage of nonzero error in the classification of benthic habitats [33]. Examples are single-beam echo sounding [34, 35], sidescan sonar (see [36] and references therein), multi-beam sonar [37, 38], aerial photography [34, 39, 40], and satellite imaging [41–43]. In each of these methods, the remote sensing data are compared to known ground survey points (the “training data”) to generate an objective or subjective model that allows prediction of the benthic habitat from those data. The result is a classification of the benthic habitat at each map point: a habitat map. From that map, the total surface coverage of target habitats, such as seagrass, are calculated and compared between monitoring events (“diachronic cartography,” e.g. [36]). These coverages, however, are not 100% accurate because different habitats can appear acoustically or visually similar (e.g. [34]). The accuracy can be estimated by collection of further ground truthing data (the “test-” or “validation data”) in which the proportions of accurate and inaccurate map points are calculated in all habitat types. Quantitative interpretation of such maps is not possible without knowledge of these errors [33], even though there are many published examples of maps created without test points to quantify their accuracy [33].

An intermediate method, remote underwater videography (RUV), is generally assumed to have the same accuracy in habitat classification as passive observations made in situ by divers, because the video sensor is deployed in the water and yields high-density full-spectrum images with video resolution on the scale of a millimeter [34, 44–51]. RUV, therefore, has been called “virtual SCUBA” and is commonly used to ground-truth habitat classification models in the above remote methods, in which workers assume that RUV has 100% accuracy in distinguishing live seagrass from algae, unvegetated surface, dead leaves, and exposed matte (a dense mixture of rhizomes, roots, and entrapped sediment; see section 3.3). Video sensors can be deployed on autonomous underwater or remotely-operated vehicles, or towed by a boat. In the last case, the camera is geopositioned by the same technology for geopositioning a sidescan sonar towfish, and depth is monitored continuously with the transducer [52]. Images can be combined into a 2D or 3D mosaic for time comparison [53, 54].

In fixed-plot methods, the same data are gathered from the same permanent field plots each monitoring event, and the temporal differences are measured directly at each plot and averaged over all the plots to obtain an estimate of the overall temporal trend [4]. Examples in seagrass monitoring include the “balisage” method of *Posidonia* monitoring within the *Posidonia* Monitoring Network (PMN) in France, and the SeagrassNet global monitoring network [55–59]. In the PMN, concrete markers are installed with iron rods along the margin of a *Posidonia* meadow and the margin position relative to each marker is photographically monitored. Other data are gathered at permanent plots adjacent to the markers, and along transects between markers. In the SeagrassNet method, iron screw anchors are installed and permanent quadrats are physically and photographically monitored along transects between the markers.

In random-plot methods, a different random sample of plots are sampled (e.g. for cover or shoot density with quadrats or transects) within the same sampling region each monitoring event. Here, the difference in habitat cover within the sampled region is not measured directly, but rather inferred from the difference in mean cover between monitoring events (for a review see [57]). Note that a critical assumption of the random-plot method is that the same fixed region is randomly sampled each monitoring event. A sampled area can be defined by GPS coordinates, or a combination of landmarks, coordinates, and depth limits.

### 2.2 Definition of decision errors

We make the simplifying assumption that monitoring consists of two observation events 0 and 1, separated by the monitoring time interval, and we are performing a simple two-sample test (paired or unpaired) to compare a descriptor of habitat health or extent, such as surface cover or seagrass shoot density. We choose this scenario because for slow-growing seagrasses such as *Posidonia*, monitoring must detect losses over short time periods in order for management intervention to be effective. Therefore, performing a trend analysis as a regression through several monitoring points may be an unaffordable luxury. For example, the EC Habitats Directive requires monitoring reports every six years, and some member states can afford one monitoring observation per reporting interval. In such cases, it is necessary to have a precise estimate of habitat loss between two monitoring events. The sampled site can be any arbitrary size; in the case of *Posidonia* monitoring programs, sites are typically defined as sea bottom with depth limits 0 and approximately 40 meters, along a segment of shoreline that can be sampled comfortably in a single working day. The results here apply to a single sampling stratum, for example the lower margin of a seagrass meadow, on a single substrate, with uniform anthropogenic impact. If the purpose of the monitoring is more complex (e.g. a before-after, control-impact BACI design with multiple spatial and temporal replicates, and hierarchical sampling), then of course many more comparisons will be made than the single stratum analyzed here, and the minimum experiment-wise sampling effort will be correspondingly larger.

There are two types of decision error in any confirmatory statistical testing [60, 61]. Type I or *α* error is the probability that a test rejects a true null hypothesis. In a monitoring context, this is the probability that we conclude there is a loss in habitat health when in fact there has been no loss. This framework defines a one-tailed test, in which we reject the null hypothesis that the difference in health between the two monitoring events (1 minus 0) is zero or positive, and we are not interested in distinguishing between zero and positive difference. The second type of error, Type II or *β* error, is the probability that a confirmatory test fails to reject a false null hypothesis. One minus *β* is then the statistical power of the test, the probability that it rejects a false null hypothesis, or that we conclude that there has been a loss in habitat in cases when there has been a true loss. We define “blind sentinel” as a method no better than a coin flip; i.e. power to detect a 10% loss is no higher than 50%. Clearly there is a wide continuum between 50% and 100%, and the goal is not a power of 50.1%. We call a method that “satisfies the 10% criterion” or “reliably” detects a 10% proportional loss, as demonstrating a power 1−*β* ≥ 0.95 of detecting such a loss in a one-tailed test with *α* = 0.05.

The above framework is a frequentist approach in which we make the conventional assumption that *α* = 0.05. There are certainly other approaches, e.g. optimizing *α* and *β* according to the relative costs of each type of error, and according to prior knowledge of the likelihood that a species is under threat, e.g. [62–67]. For example, there is growing evidence that *Posidonia oceanica* is currently experiencing unacceptable declines throughout the Mediterranean Sea [32], which suggests that we have finally reached the point where investment in recovery efforts might be generally more beneficial than investment in monitoring efforts, or that our default assumption should now be that the species is declining at any site in the absence of strong evidence otherwise. Nevertheless, because of the near-universal convention of setting *α* = 0.05 or smaller, we will make this assumption. This is a conservative choice because the resulting minimum sample sizes will still apply under alternative approaches with *α* > 0.05, and our comparisons of methods are valid regardless of the magnitude of *α*. Nevertheless, we agree with e.g. [62, 63] that a cultural change is necessary in the use of Type I error in monitoring and environmental impact studies.

### 2.3 Statistical power relationships

In the simple case of a *t*-test, with minimum detectable difference *δ* for a power of 1−*β*, a Type I error of *α* in a one-tailed test, and a standard deviation *s*_*md*_ of the mean difference in the descriptor between the two monitoring events, the power of the test is

$$\text{power}=P[T\left(\delta /s_{md}\right)>t_{1-\alpha }],$$ (1)

where *T*(*δ*/*s*_*md*_) is a *t* variable with non-centrality parameter *δ*/*s*_*md*_, and *t*_1−*α*_ is the critical *t* value for a one-tailed test at the significance level *α*. In this paper all calculations of power, minimum detectable difference, and minimum sample size are made from Eq 1 using the functions power.t.test() or power.prop.test() in R 3.0.2 [68]. The first function applies to a *t*-test on means of a continuous variable, and the second to a chi-square test on proportions.

To a very good approximation (Eq 7.11, p. 117 in [69]),

$$\delta \;≅s_{md}\left(t_{1-\alpha }+t_{1-\beta }\right),$$ (2)

where *t*_*p*_ is *t*-value yielding a cumulative (left) probability equal to *p*. For large sample sizes, *α* = *β* = 0.05, and a one-tailed test,

$$\delta \;≅s_{md}\left(2\right)\left(1.645\right)=\left(3.29\right)\left(s_{md}\right).$$ (3)

These Eqs (2 and 3) can be used in any context without the need to compute probabilities from the non-central *t* distribution.

The value of *s*_*md*_ depends on the sampling method. If *n* random plots within the same sampling region are sampled independently each monitoring event, then

$$\begin{array}{c} s_{md}=s\sqrt{2/n}, \end{array}$$ (4)

where *s* is the standard deviation in the habitat descriptor pooled across the two monitoring events. This is composed of spatial variation in the descriptor and variation inherent the methodology itself. If the same *n* fixed plots are sampled each monitoring event, then

$$\begin{array}{c} s_{md}=\frac{s_{d}}{\sqrt{n}}, \end{array}$$ (5)

where *s*_*d*_ is the standard deviation in the difference in the descriptor within a fixed plot between the two monitoring events [69]. In all cases *s*, *s*_*d*_, and *s*_*md*_ may be expressed relative to the mean of the descriptor, so that these are interpreted as coefficients of variation rather than as standard deviations, and *δ* is then the minimum detectable proportional difference, and our target is a minimum detectable proportional difference of 10% or less.

In practice, a two- or single-sample *t* test might not be preferred over a non-parametric equivalent, because of the details of the data residuals. In these cases, the statistical power might be somewhat greater or lower than in Eq 1.

### 2.4 Direct sampling of random or fixed plots

The last two equations show that, assuming large sample size, the fixed-plot method is statistically more powerful than the random-plot method whenever

$$\begin{array}{c} s_{d}<s\sqrt{2}≅1.41s. \end{array}$$ (6)

We now consider the implications of this result for direct (SCUBA) sampling of habitat cover or seagrass shoot density.

Within the random plots design, shoot density is sampled by placing a 40×40-cm quadrat randomly on the ground within a fixed sampling region, and painstakingly counting by hand each shoot that originates within that quadrat, taking care to count every shoot and not count any shoot twice [57, 70]. In *Posidonia* the most informative measures are taken near the lower margin of a seagrass meadow, since the lower margin is expected to experience the greatest regression immediately in response to slightly lowered water quality, while higher regions of the bed may not visibly respond immediately or at all. The sampling region at the lower margin might be defined by depth isobars parallel to shore, between landmarks or GPS coordinates. Both present and absent patches are sampled for cover or shoot density, with absent patches representing a data point of zero.

We can calculate the minimum possible standard deviation in shoot density by noting that variation in shoot density is the sum of two contributions: the contribution due to seagrass presence/absence, and the contribution due to variation within patches containing seagrass. If we consider only the first contribution, then the resulting standard deviation is an underestimate. In a patchy meadow where mean seagrass cover is *p*, the standard deviation in cover is approximated by the Bernoulli standard deviation of a point estimate, $\sqrt{p(1-p)}$[69]. This is equal to a coefficient of variation (CV) of one in a patchy meadow with *p* = 0.5, which is common near the lower margin of a *Posidonia* meadow where monitoring is critical (see section 3.1). Then in such a meadow, the spatial CV in shoot density is at least 1, and will be indefinitely higher because of the added variance among patches with *Posidonia* present. Then the CV in the mean difference in shoot density between monitoring events is $s_{md}/p=CV_{md}>\sqrt{2/n}$, where *n* is the number of independent quadrats each monitoring event. The actual CV in shoot density could be higher by orders of magnitude; e.g. in a region of positive skew, where the majority of plots are low density and there are a few plots of high density.

**Fig 1:**
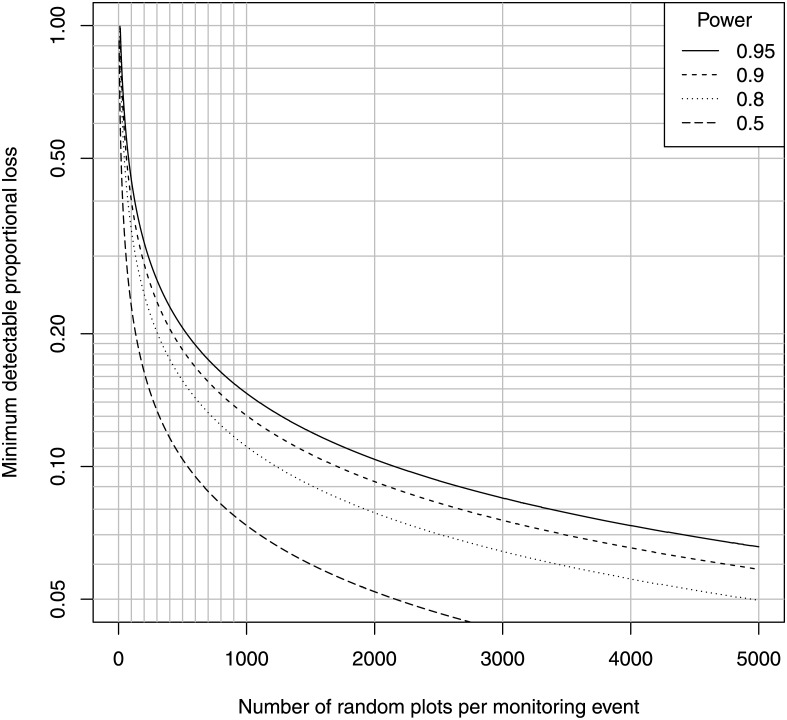
Minimum detectable difference, random-plot design. Minimum detectable difference in habitat cover for the indicated number of random plots per monitoring event and power for detection of the difference in a two-sample, one-sided *t*-test, *α* = 0.05.

Substituting $\sqrt{2/n}$ for *s*_*md*_ into Eq 1 gives Fig 1, the minimum detectable proportional difference in shoot density using a random plot method for a range of values of *n*, the number of independent quadrats per monitoring event, and the indicated values of power.

Fig 1 shows that the minimum number of quadrats for the detection of a loss of 10% (from a cover of 0.5 to 0.45) exceeds 2000 for a statistical power of 0.95. A blind sentinel method results from any sampling effort lower than about 500. Note that in a patchy meadow with cover = 0.5, half these sampling points are expected to have a value of zero. This result applies only to a single sampling stratum per monitoring event, e.g. along the lower margin of the bed on a uniform substrate. Additional sampling is necessary for each additional stratum. The number of sampling quadrats per sampling site used in practice to monitor shoot density in *Posidonia* rarely exceeds 36 (see also section 3.1, [29]). As shown in Fig 1, this sampling effort cannot reliably detect a loss less than about 50%. Conversely, this sample size can detect a 10% loss with probability only 0.10 (*α* = 0.05 in a one-sided test), a substantially worse perfomance than a coin toss.

In a fixed-plot method for monitoring the location of the margin of a seagrass meadow, or shoot density along the margin, the difference in the descriptor is measured directly rather than inferred relative to the large spatial variance among random plots each monitoring event. The standing spatial variance in cover or shoot density is thus irrelevant, and the only relevant variance is that of the difference within each plot between monitoring events.

The great advantage of the fixed-plot method lies in the fact that if the causes of habitat loss are diffuse, as is expected in an aquatic environment where a decline in water quality affects all plots about equally, then there may be essentially no variance among fixed plots in the loss they experience. In this case, the standard deviation of the difference in Eq 5, is just the sum of the measurement errors inherent in the method, which by definition will satisfy Eq 6 if the same measurements are made in a random plot method. If the margin position is measured to within a few centimeters precision, and the fixed plots are reasonably stable and do not shift more than a few decimeters during the monitoring interval, then the *s*_*d*_ in Eq 5 might be on the order of a few decimeters, and the minimum detectable mean margin regression for a sample size of 20 fixed plots will be roughly $3.29\times 30/\sqrt{20}=22$ cm. This regression is less than one hundredth the surface area of any long fringing meadow of width 22 meters or more, if the regression occurs along the long dimension of the meadow. Thus, if the causes of *Posidonia* loss are diffuse and equal throughout such a meadow, then a fixed-plot method can detect one-tenth the loss of a random plot method with one hundredth the sample size.

The situation becomes a bit more complicated if we relax the assumption that the disturbance is diffuse in the water column, but rather spatially variable, as is expected for example by mechanical damage at unpredictable locations. In this case we can calculate the maximum possible variance among fixed plots if we assume values for the maximum possible increase and decrease in the descriptor within each plot. For example, if our descriptor is the position of the lower margin of a *Posidonia* bed, then the maximum possible progression is the maximum annual linear growth of *Posidonia*, if our monitoring interval is one year, plus the maximum possible chance movement of the fixed plots in one monitoring event. The maximum allowable regression is an a priori decision: we simply decide that if the regression is greater than some threshold at any fixed plot, then this is an “easy” case where no statistical analysis is necessary, and we assume that this regression is great enough that the disturbance that causes it ipso facto warrants a management intervention. Then we are concerned only with “difficult” cases where all instances of regression at fixed plots is lower than this maximum, and therefore statistical analysis is necessary to test for a mean difference across all plots.

**Fig 2:**
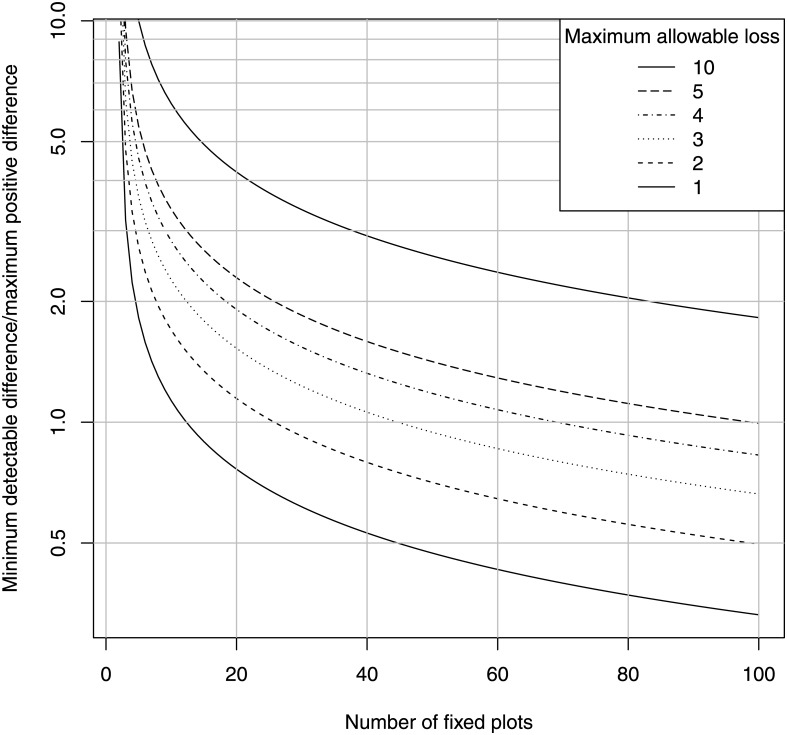
Minimum detectable difference, fixed-plot design. Minimum detectable difference in habitat cover for the indicated number of fixed plots per monitoring event and power for detection of the difference in a paired, one-sided *t*-test, *α* = *β* = 0.05.

The maximum variance in the fixed-plot difference then occurs with half the plots exhibiting the maximum progression and the other half with the maximum regression during the monitoring interval. By definition this variance is

$$\begin{array}{c} s_{d}^{2}=l_{m}\left(l_{m}/4+1/2\right)+1/4, \end{array}$$ (7)

where *l*_*m*_ is the ratio of maximum regression to maximum progression. Substituting this into Eq 1 gives Fig 2, the minimum detectable loss for arbitrary *n*, the number of fixed plots at each monitoring event.

For example, Fig 2 shows that if the maximum progression of *Posidonia* is 0.3 meter (which includes both linear growth of the seagrass plus chance movement of underwater markers), and the maximum allowable regression is five times this maximum progression, then for 20 fixed plots our minimum detectable mean loss is between 2 and 3 times the maximum progression, which comes to 0.6 to 0.9 meter of regression, for a power of 0.95. So for 20 fixed balisage plots that are reasonably stable, we will detect a mean regression of less than one meter at the lower margin of a *Posidonia* meadow very reliably, in the worst case scenario where the disturbance is not diffuse. This amounts to roughly five times the detectable regression if the loss disturbance is diffuse in the water column, but is still achieved at most at one hundredth the minimum sampling effort of the random plot method. Another way of arriving at this result is via Eq 6: if *l*_*m*_ = 5, then the standard deviation in the difference is 3. In *Posidonia oceanica*, this *s*_*d*_ is equivalent to roughly 70 cm of margin regression, which constitutes one hundredth of the cover of a long fringing meadow of width 70 m (see above). Hence *s*_*d*_ ≅ 0.01 in units of proportional cover in such a meadow, while *s*_*md*_ = 0.5 in units of proportional cover; hence *s*_*d*_ ≅ 0.02 *s*_*md*_, easily satisfying Eq 6. This result clearly demonstrates a 70-fold superiority of the fixed-plot method in units of *δ* for such a meadow.

Note however that Fig 2 assumes that the fixed plots are not lost due to unstable substrate or strong currents between monitoring events; the number of fixed plots will need to be increased to compensate for any loss or chance movement greater than a few decimeters.

### 2.5 Remote visualization methods

#### 2.5.1 Remote underwater video (RUV)

The above analysis recognizes that in the random-plot method, *s* in Eq 4 is large and fixed because it is the natural spatial variation in cover and shoot density within the fixed sampling region. Hence, within the random-plot method, the only way to decrease *s*_*md*_ (and thereby increase power) is to increase the sample size *n*. This is difficult if the only method for increasing the sample size is to increase the number of quadrats or transects taken during SCUBA dives, with its limits in cost, time, and fatigue. However, a remote method can increase the rate of observation of sea bottom by orders of magnitude over SCUBA, thereby achieving the sampling intensity required by Fig 1. The remote method that is most similar to direct observation is remote underwater video (RUV), which is generally assumed to have zero error in distinguishing live seagrass from exposed matte, algae, or unvegetated surface, and hence requires no ground truthing for verification. This method allows estimation of spatial cover rather than shoot density; only the former is required for habitat monitoring under the EU Habitats Directive.

As Fig 1 shows, a sample size of over 2000 random points per study site is necessary for reliable detection of a 10% loss in cover. Can RUV achieve this sampling intensity? RUV can generate sea-bottom images at a rate of 1–5 square meters per second, depending on the elevation of the sensor above the bottom and water clarity. This represents at least three kilometers of > 1-meter belt transects per hour of field time. If the sampling site is a 1-km segment of coastline, RUV could run three parallel transects the entire length of that coastline per hour. In contrast, SCUBA transect methods for estimating cover of live *Posidonia* can achieve approximately 50 meters of transects at three random locations within that field site per field day, which amounts to roughly a 400-fold difference. Making the conservative assumption that 5 meters of transect length is at least equivalent to one random sampling point, then RUV can achieve at least 600 sampling points per hour, and at least 3600 in one six-hour field day, which is sufficient to achieve the 10% criterion in Fig 1 at a power of 0.95, for any segment of coastline of length about 1 km or smaller. Note that here GPS is absolutely unnecessary to renavigate previous transects, because this is a random-plot design and transects are different and random each monitoring event. Accurate GPS, however, may be necessary for delineating the boundaries of the fixed sampling region, but this is true for any method of seagrass monitoring.

This power can in theory be increased by using a fixed-plot method, in which submeter-accuracy, differential GPS is used to navigate close to the same fixed transects each monitoring event [49]. Such navigation will increase power above the random-plot power whenever seagrass patch radius is equal to or greater than GPS error, otherwise power will never be lower than for a random-plot method [49]. RUV thus is the only method considered here that can satisfy the 10% criterion within either a random- or fixed-plot sampling regime, and without any requirement for people underwater.

#### 2.5.2 Classification error creates bias

The main source of statistical error in the remaining remote or indirect methods is the error in classifying the substrate. This error is inherent in all acoustic methods and visual methods where the sensor is above water, because loss of information in the signal creates classification ambiguity. In this section we explore the effect that classification error has on the statistical power of these methods used purely for monitoring habitat cover.

We assume that we are interested only in measuring the proportional loss of habitat between two monitoring events. Therefore, we hope to classify habitat presence as presence, and habitat absence as absence, at each monitoring event. However, our accuracy will never be 100%, and therefore two kinds of classification error exist. Defining these errors in terms of seagrass habitat, the false-positive error *e*_*p*_ is the conditional probability of classifying a habitat point as seagrass, given that it is non-seagrass, and the false-negative error *e*_*n*_ is the conditional probability of classifying a point as non-seagrass given that it is seagrass. The precise values of these error rates will depend on the mixture of habitat types at a given location, since they are a weighted average across habitats.

To see how classification error affects monitoring results, we first divide all ground habitats into seagrass and non-seagrass, and assume that the true proportion of the map that is seagrass at times 0 and 1 is *p*_0_ and *p*_1_. We then imagine a classification model that converts the acoustic or visual data at each map point to habitat type. The output of this model yields an observed proportion of seagrass at all map points at times 0 and 1 equal to *o*_0_ and *o*_1_. We can now express the observed seagrass proportion in terms of the true proportions at either monitoring event [33, 71]:

$$\begin{array}{l} o=p\left(1-e_{n}\right)+e_{p}\left(1-p\right)\\ \;=p\left(1-e_{n}-e_{p}\right)+e_{p};\;\text{therefore}, \end{array}$$ (8)

$$p=\frac{o-e_{p}}{1-e_{n}-e_{p}}.$$ (9)

This equation shows that in the presence of classification error of either type, the observed proportion is biased upwards or downwards depending on the magnitudes of the classification errors and the true proportion of the target habitat. For example, if there is no seagrass (*p* = 0), then the observed seagrass cover is the false positive error, an overestimate. If true cover is *p* = 1, then the observed cover is one minus the false negative error, an underestimate. If the two classification errors sum to 1, then the observed cover is always equal to the false positive error. Generally, the observed cover will be an overestimate for low *p* and an underestimate for high *p*, and the bias increases with increasing classification errors. For example, assume *p* = 0.2, and the false negative error is 0.1. Then 0.18 of all map points will be true seagrass classified as seagrass. If the false positive error is 0.8, then an additional 0.64 of all map points [(1−0.2)×0.8] will be falsely classified as seagrass. Then our total estimated seagrass cover will not be 0.2, but 0.18 + 0.64 = 0.82. In general, the largest bias results when the habitat with the highest classification error dominates, and these errors can move the bias in either direction: upwards if non-seagrass and false positive error are high, and downwards if seagrass and false negative error are high.

However, even though classification errors exist, in cases where the errors are identical each monitoring event we might be able to simply ignore them and assume that the errors cancel out when we calculate the difference in the target habitat between the two monitoring events. This unfortunately is not the case. From the above equations,

$$o_{1}-o_{0}=\left(p_{1}-p_{0}\right)\left(1-e_{n}-e_{p}\right);\;\text{therefore},$$ (10)

$$\frac{o_{1}-o_{0}}{p_{1}-p_{0}}=1-e_{n}-e_{p}.$$ (11)

This shows that the ratio of observed to true difference in the target habitat is discounted by the sum of the two classification errors, that each error has the same effect, and the estimated difference might actually be the reverse of the actual difference if the classification error is high enough. For example, assume that a seagrass bed is destroyed between monitoring events, with the cover falling from 1 to 0, from a continuous meadow to a habitat of macroalgae on gravel. Also assume an acoustic map with false negative error of 0.2 and false positive error of 0.8 for algae on gravel. Then at time 0 we would estimate a cover of 0.8, and at time 1 we would estimate a cover of 0.8, and conclude there was no change, when in fact the entire meadow was lost. Indeed in this example, we will always observe a cover of 0.8 regardless of the true starting or ending values of cover, *because* the classification errors are constant.

#### 2.5.3 Variance in classification error creates uncertainty

Nevertheless, there are many published studies of changes in bottom habitat in maps based on classification models in which the habitat coverages are not corrected by the classification error using Eq 9 (see [36] and references therein). This raises the question: under what circumstances can these maps be considered reliable monitoring tools; especially, reliable enough to satisfy the 10% criterion? The answer, as discussed presently, is that the classification error and its variance both must be close to zero. Here we consider the possibility that classification error can vary between monitoring events, and influence the uncertainty in map estimates of habitat cover.

Certainly there are many reasons why the false positive or negative errors will change between monitoring events: the habitat matrix might change, thereby changing the error rates; the precise conditions under which the acoustic or photo image was taken will be different and therefore with different resulting data even though the habitats might be unchanged; geopositioning error introduces classification error along the margins of continuous ground habitats or everywhere in a patchy matrix. Therefore, an observed loss or gain in any habitat may reflect nothing more than a change in classification errors between monitoring events. For example, consider a site where the true seagrass cover declines from 0.5 to 0.45 between monitoring events (a 10% loss), the false negative error changes from 0.2 to 0.05, and the false positive error changes from 0.05 to 0.2. Then the observed seagrass cover will be 0.43 at the first monitoring event and 0.54 at the second, which represents an observed increase of 26%. This is 2.6 times the actual change and in the opposite direction, and is entirely an artifact of the change in classification error. Variation in classification error clearly represents random noise that can obscure any real change or constancy in habitat cover.

To propagate variation in classification error to variation in the estimated difference of habitat cover between two maps, we first take the variance of both sides of Eq 10, treating each classification error as a random variable:

$$\text{var}\left(o_{1}-o_{0}\right)=\text{var}[\left(p_{1}-p_{0}\right)\left(1-e_{n}-e_{p}\right)]$$ (12)

$$=\text{var}\left(e_{n}\right)\left(p_{0}^{2}+p_{1}^{2}\right)+\text{var}\left(e_{p}\right)[{\left(1-p_{0}\right)}^{2}+{\left(1-p_{1}\right)}^{2}].$$ (13)

**Fig 3:**
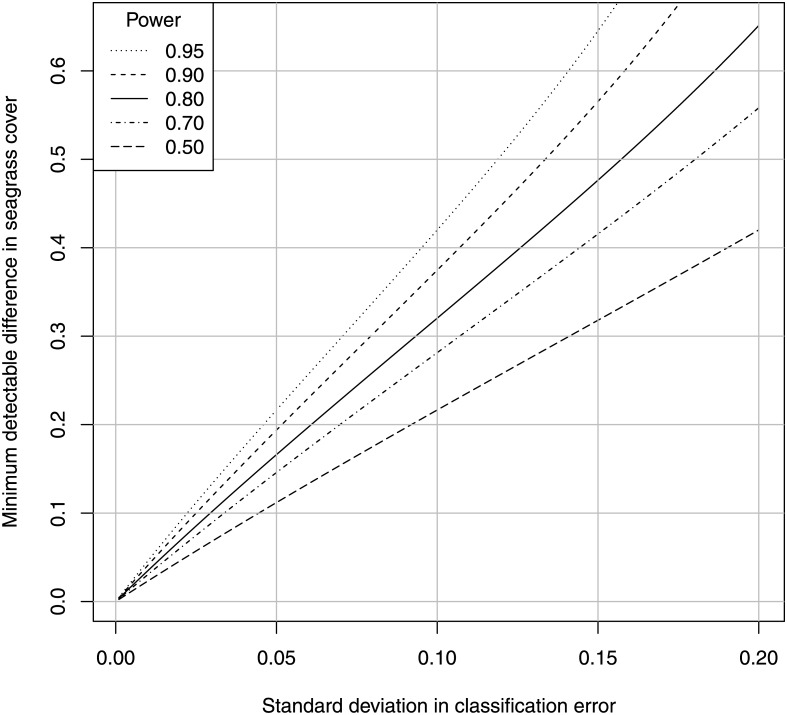
Minimum detectable difference between two maps with variance in classification error. Minimum detectable difference for the indicated standard deviation in positive and negative classification error, for the indicated power in a comparison between two maps (*α* = 0.05, see section 2.5.3).

For arbitrary variance in classification errors, we can thus calculate the variance in the observed difference in habitat cover between two maps, the margin of error of this difference, and the minimum detectable proportion difference (from Eq 1), with detection defined as the margin of error not overlapping zero. For the indicated standard deviation in classification error, Fig 3 provides this information, with the margin of error equal to the minimum detectable proportional difference for power = 0.5.

Here we see a very simple result, namely that if the standard deviation in both classification errors is *e*, then the standard error of the observed difference in habitat cover is a bit larger than *e*, the margin of error for this difference is a bit larger than 2*e*, and the minimum detectable difference (for a power of 0.95) is approximately 4*e*. Thus, if the standard deviation in classification error is greater than 0.05, then the method is a blind sentinel. On the other hand, if the standard deviation is less than 0.025, then the method is capable of satisfying the 10% criterion.

Field workers can use this figure to report the error bars around any habitat difference calculated between two maps. For example if a difference is observed to be 0.1, then this would be reported as *d* = 0.1±0.2 for the 95% confidence interval, assuming that classification errors themselves have a standard deviation at most 0.1. If the true observed proportional difference were 0.4, then the probability is 95% that the above confidence interval will not overlap zero.

The above analysis clearly demonstrates that the difference in habitat cover between two uncorrected habitat maps is a blind sentinel method unless the standard deviation in classification error is near zero. Such uncorrected maps may, however, be able to detect losses as low as 30–50%, if the methods are careful and meticulous enough to maintain the standard deviation at 0.1 or below. Note, however, that we have ignored the bias in cover estimation caused by classification error, and this is an additional problem with uncorrected maps over and above the variance in classification error. This appears to be an insoluble problem, since the only way to be confident of a low classification error and its variance is to estimate the classification error, which requires field work that might negate the main advantage of a remote method.

#### 2.5.4 The utility of habitat maps corrected by classification error

The above results indicate that changes in habitat cover estimated from classification models may be meaningless (due to both biases and propagation of classification variance) unless classification error is explicitly measured and used to correct the estimate of habitat loss. If we know the false positive and negative errors *e*_*p*0_, *e*_*p*1_, *e*_*n*0_, and *e*_*n*1_, then we can use these to create an estimate of the true habitat change *p*_1_−*p*_0_ from the observed change *o*_0_−*o*_1_, by rearrangement of Eq 10:

$$\hat{p_{1}-p_{0}}=\hat{d}=\frac{o_{1}-e_{p1}}{1-e_{n1}-e_{p1}}-\frac{o_{0}-e_{p0}}{1-e_{n0}-e_{p0}},$$ (14)

where $\hat{d}$ is the estimated true difference in habitat cover between the two monitoring events, corrected by the measured classification error. Here we no longer assume classification errors are equal during the two monitoring events: they can be arbitrarily different, because now we have measured their values and use them to explicitly correct the bias.

Exact measurement of their values would require that we directly observe the habitat in the field represented by every point on the map, and calculate the overall proportion of correct and incorrect classification of every point in both monitoring events. Obviously this would defeat the purpose of remote mapping. Instead, of course, we observe the ground habitat at a practicable random sample of map points in target and non-target habitat, and use the observed proportion of incorrect classifications as an estimate of the true classification error across the entire map. This estimate, however, has a sampling error, and this sampling error will be propagated to our corrected estimate of true habitat loss above. This raises the question: how does this sampling error affect the map estimate of habitat loss?

The variance of the map estimate in Eq 14 is the variance of the difference of two ratios, which is equal to the sum of the variances of the ratios. The variance of the ratio of two random variables *x*/*y* can be approximated as ([72], p 351):

$$\begin{array}{c} \text{var}\left(x/y\right)=\frac{\mu_{x}^{2}}{\mu_{y}^{2}}[\frac{\text{var}(x)}{\mu_{x}^{2}}-2\frac{\text{cov}(x,y)}{\mu_{x}\mu_{y}}+\frac{\text{var}(y)}{\mu_{y}^{2}}], \end{array}$$ (15)

with an error on the order of the reciprocal of the sample size. Here we assume that the variance of the estimates of the classification errors is just the binomial variance of a proportion, that the sampling variances of each classification error are independent of each other, and these are the only sources of variation in the computation of the true map difference:

$$\begin{array}{ll} \mu_{x} & =1-e_{p},\\ \mu_{y} & =1-e_{p}=e_{n},\\ \mathrm{var}(e_{p}) & =\sqrt{e_{p}(1-e_{p})/n},\\ \mathrm{var}(e_{n}) & =\sqrt{e_{n}(1-e_{n})/n},\\ \mathrm{var}(x) & =\mathrm{var}(o-e_{p})=\mathrm{var}(e_{p}),\\ \mathrm{var}(y) & =\mathrm{var}(1-e_{p}-e_{n})=\mathrm{var}(e_{p})+\mathrm{var}(e_{n}),\;\text{and}\\ \mathrm{cov}(x,y) & =\mathrm{cov}(o-e_{p},1-e_{n}-e_{p})\\ & =\mathrm{cov}(e_{p},e_{n}+e_{p})=\mathrm{var}(e_{p}). \end{array}$$

**Fig 4:**
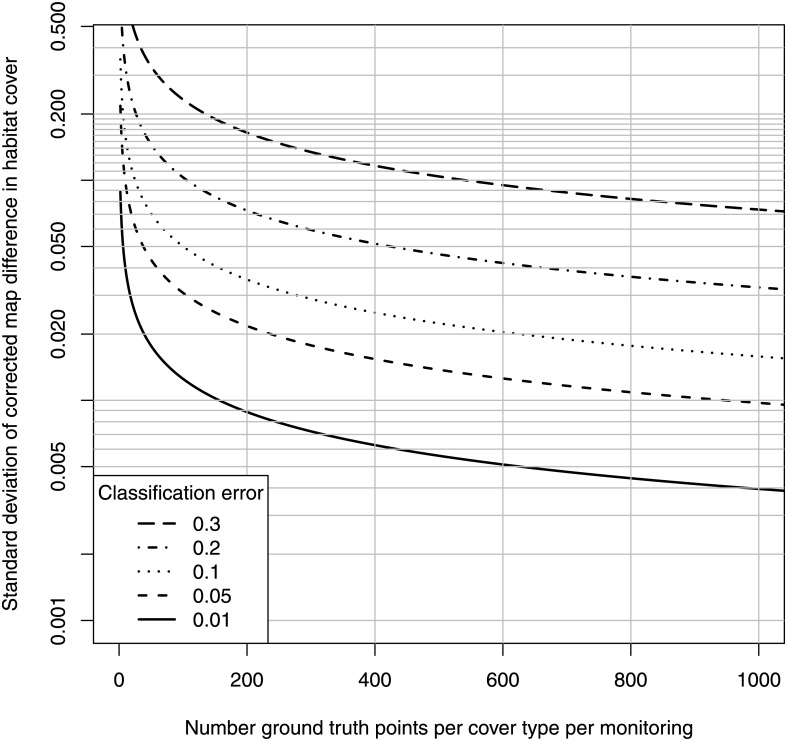
Standard deviation of corrected map difference as function of number of ground truth points. Standard deviation of corrected map difference as a result of sampling error of ground truthing with the indicated number of ground truth points, and indicated positive and negative classification error.

**Fig 5:**
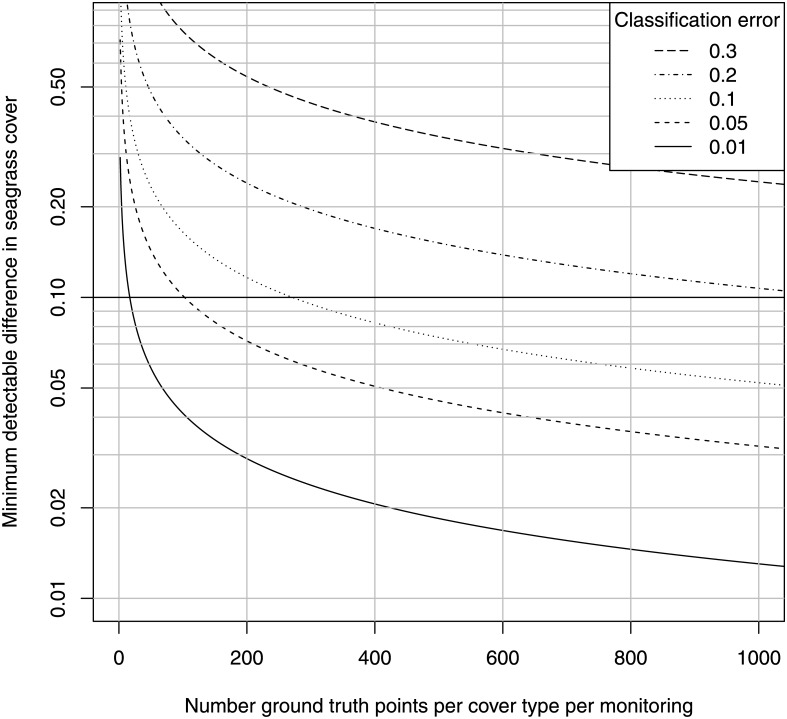
Minimum detectable corrected map difference as function of number of ground truth points. Minimum detectable corrected map difference as function of number of ground truth points, for indicated positive and negative classification error (*α* = *β* = 0.05).

**Fig 6:**
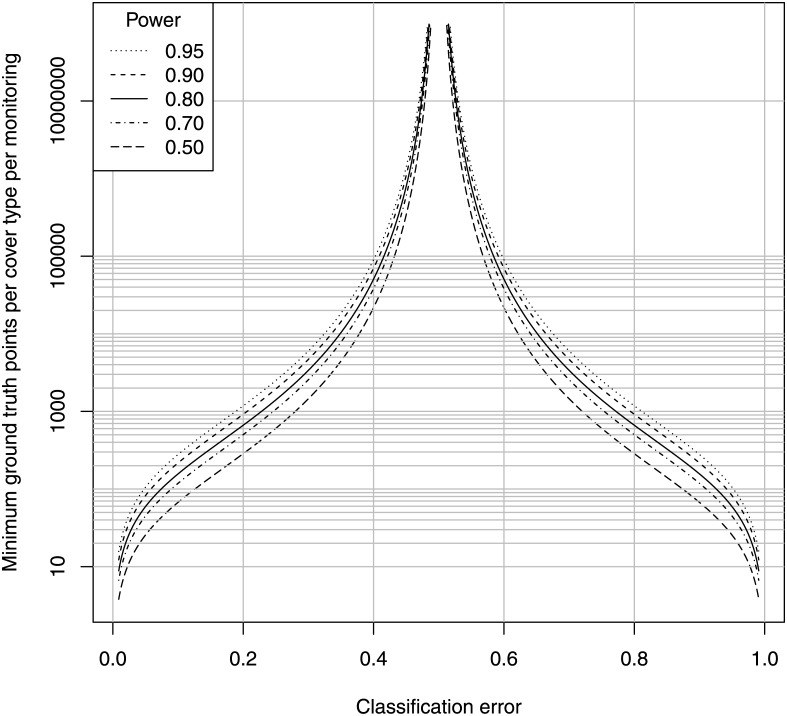
Minimum ground truth points to detect 10% loss in corrected map. Minimum number of ground truth points needed to correct a map to sufficient precision to detect a 10% loss, for the indicated power and classification error (*α* = 0.05).

Substituting these expressions into Eq 15 and taking the sum of the variance of the two ratios in Eq 14 gives the variance in the corrected map habitat loss. The square root of this variance is plotted against the number of ground truth points per habitat (seagrass and non-seagrass) for the indicated actual classification error in Fig 4. For example, if our positive and negative classification error is 0.2, and we estimate these errors by sampling a random 100 ground points in both seagrass and non-seagrass, for a total of 200 points each monitoring event, then the standard deviation of the corrected map difference in seagrass cover is 0.1, and the margin of error would be (1.96)(0.1) = 0.2; Fig 5 shows that this sampling effort translates to a minimum detectable difference in cover of about 0.4. Fig 6 shows the minimum number of ground truth points per habitat necessary to detect a map difference of 0.1, for the indicated power and classification error.

This figure shows that in the range of commonly reported lower limits of *Posidonia* classification error, e.g. from 0.2 to 0.35 (see section 3.4), from nearly 2000 to over 10000 ground truth points per habitat per monitoring event are necessary to achieve a corrected map difference that is precise enough to reliably detect a 10% loss, and any sampling effort with fewer than 500 to 3000 ground truth points is a blind sentinel method. Note that this minimum sampling effort increases without limit as classification error approaches 0.5. This is because, at a classification error of 0.5, the map is no better than a coin toss and therefore contains no information, and cannot be corrected regardless of the sampling effort. Note also the symmetry of this Figure: a classification error of 1−*e* has exactly the same information value as a classification error of *e*.

These results raise a final question. Because the ground truth study needed to correct the map is the direct observation of the ground at numerous georeferenced points, and therefore constitutes valuable data that can be used for an immediate estimate of habitat cover that has zero classification error, why not use these data to estimate seagrass habitat cover directly, rather than go to the extra effort to create and attempt to correct an error-laden map that provides only an indirect estimate?

**Fig 7:**
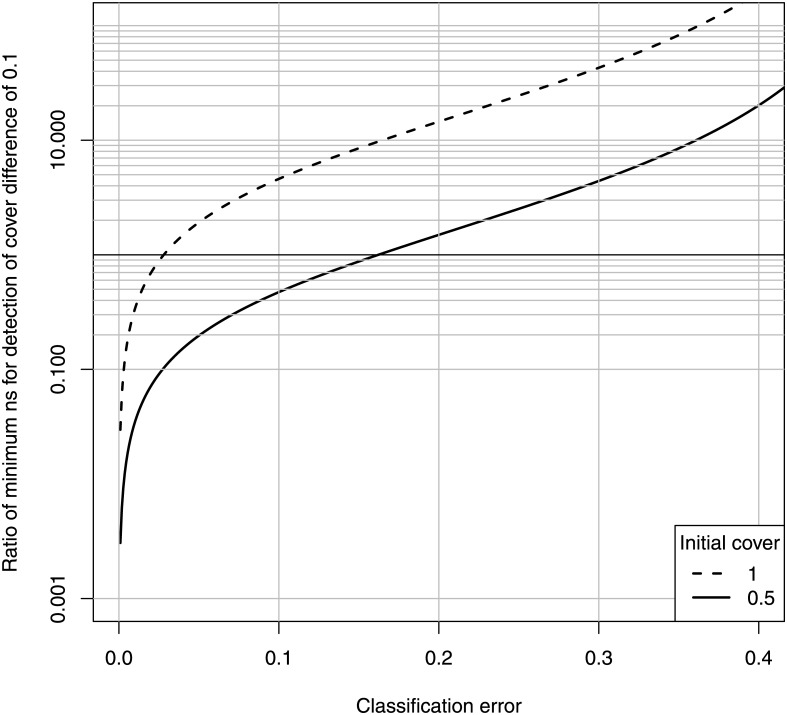
Ratio of minimum ground truth points for map correction to minimum ground truth points for direct estimate of 10% loss. Ratio of minimum ground truth points for map correction to minimum ground truth points for direct estimate of 10% loss, for the indicated classification error and initial (seagrass) habitat cover (*α* = *β* = 0.05).

We answer this question by comparing the minimum ground truth sample size for estimation of a 10% map loss to that for estimation of a 10% loss directly by random point sampling. The latter is a simple chi-square test of a difference between two proportions: the proportion of ground truth points containing the target (seagrass) habitat at the two monitoring events. In this proportion test, the standard deviation of the difference in proportion to be substituted into Eq 1 is simply $s_{md}=\sqrt{2/n}$, where *n* is the number of ground truth points in each habitat. Taking the ratio of the minimum ground truth points for detecting a 10% loss after map correction, to the minimum for detecting a 10% loss directly by comparing the observed covers, gives Fig 7.

This figure shows that, for example, if the classification errors are each equal to 0.2, then the minimum number of ground truth points necessary to detect a 10% loss after map correction is from 1.5 to 15 times the number needed to directly detect a 10% loss, depending on the starting habitat cover.

**Fig 8:**
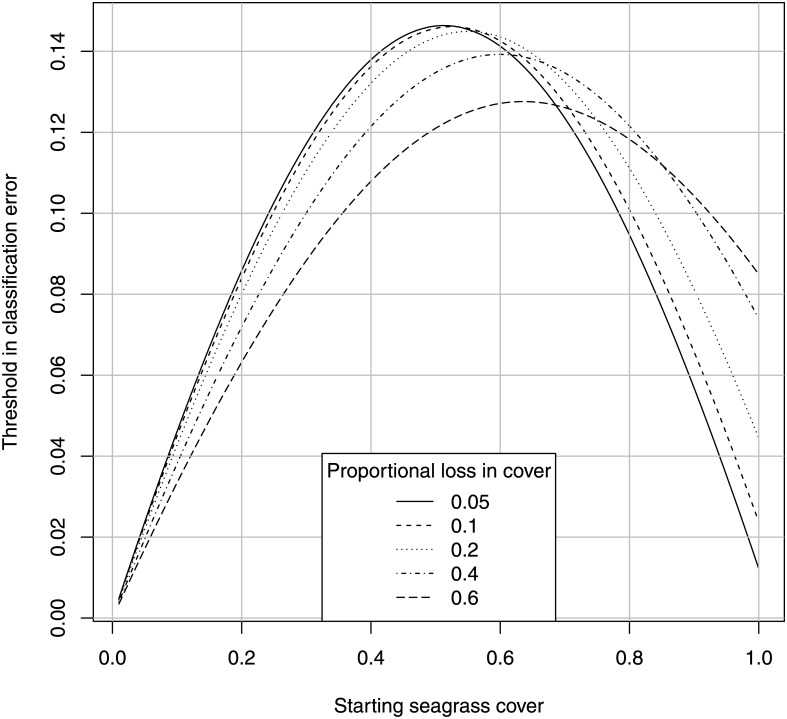
Threshold in classification error at which field labor is equalized for estimating 10% loss. Threshold in classification error at which field labor is equalized for direct versus map estimation of cover loss, for the indicated values of starting habitat cover (*α* = *β* = 0.05).

Fig 8 shows this relationship from a different perspective, the threshold classification error at which the field labor is equalized: the labor for estimating a 10% loss directly is equal to the labor for estimating the loss from two maps. This we call the “utility threshold” of a map habitat classification for sentinel monitoring. Values above the lines favor the direct method, and below the lines favor the map method. This figure shows how this threshold depends on the starting habitat cover, with the maximum near the maximum binomial variance of *p* = 1/2.

The reasons that map error is often more difficult to estimate than habitat cover directly are 1) two probabilities must be estimated rather than one (the positive and negative error rates); and 2) as classification error increases, the information contained in the map decreases, and the minimum sample size to correct the map then increases exponentially to infinity at a map error of 0.5.

Note that these two figures undervalue the direct estimation method, because we assume that the only field effort in the map method is that required to validate the map. In reality, some unknown number of training points will also be necessary. If the number of training and test points are equal, then the ratio is double in Fig 7, and the utility threshold is half that in Fig 8. Note also that a better sampling design for directly estimating habitat loss is a fixed-plot design. This however, would require the construction of permanent underwater markers, which would defeat the purpose of a remote mapping method. If such markers are present, then they would provide a direct estimate of seagrass loss at probably 1/100th the field effort required to quantify the classification error of the map. Thus, Figs 7 and 8 are meaningful only if a random-plot method is used for the direct estimate. A well-designed fixed-plot method is superior to all maps other than those known in advance to have a classification error essentially equal to zero.

These results indicate that remote mapping methods are a misnomer in that they require substantial field effort to correct for classification error, and if the error is not well below the utility threshold of 0.15, then remote mapping methods require far more labor to validate the map than would be needed measure overall habitat loss directly.

## 3 Discussion

The success of a sentinel monitoring program hinges on its ability to detect when a resource is in decline. To do this, monitoring must distinguish between chance variation and true decline. This ability, namely statistical power, has been neglected in the design of environmental monitoring programs worldwide, and specifically programs to monitor health of seagrass communities, which contribute ecosystem services in excess of one million euros per hectare per annum [5, 8, 13]. Some workers have proposed a minimum performance criterion of 10% for seagrass monitoring programs: a management goal should be to develop and use only programs powerful enough to detect a true proportional loss of 10% [10]. Especially in slow-growing species such as *Posidonia oceanica*, timely detection of small losses is critical [11, 12].

Here we have reviewed the expected statistical power of common habitat monitoring methods, using both empirical knowledge of the seagrass *Posidonia oceanica* and basic statistical concepts, to ask which methods are and are not capable of satisfying this 10% criterion. Below we discuss the significance of each of our major results.

### 3.1 Random-plot direct methods for monitoring seagrass cover or shoot density are generally blind-sentinel methods

Random-plot direct methods for sampling seagrass cover and shoot density are attractive because they are conceptually simple, do not require the construction of permanent markers or specialized technology or expertise, and can be carried out by largely untrained volunteer labor such as SCUBA dive clubs, e.g. [73]. Unfortunately, however, these methods have a critical disadvantage: all standing spatial variation in the response variable is incorporated into the residual error term of the statistical test. As [4] p. 85 remarks, “re-randomization does nothing more than cloud the comparison of differences, without truly adding error degrees of freedom.” Where such standing variation is high, this is an enormous handicap, especially in cases where direct methods require SCUBA, with its attendant costs, risks, and field limitations.

In the case of *Posidonia* and many other seagrasses, cover and shoot density are highly sensitive to natural microhabitat variation, and therefore are highly variable at all spatial scales, as has been shown in many studies [57, 71, 75]. Patchy distribution of live *Posidonia* and dead matte is natural and common especially near the lower depth limit of meadows, where monitoring is critical. Such high variation is ironically documented and presented in field reports of random-plot seagrass monitoring methods. For example, in the final field test for the national *Posidonia* monitoring protocol for Croatia, Guala et al. [75] find that the CV in shoot density within *Posidonia* patches ranges from 40% to 60% near the lower depth margin at several field sites in their pilot demonstration of the protocol, and cover of live *Posidonia* is close to 50% at the lower margins. This high variation requires a minimum of 2000 independent quadrat samples per monitoring event to reliably detect a 10% loss in shoot density using a random-plot design (Fig 1). Yet the sample size for random-plot methods of monitoring shoot density in *Posidonia* mandated programs is 33 to 36 quadrats per sampling site, fewer than two percent of this minimum [29]. Even this tiny sample size is inflated, however, because in reality most samples are subsamples of only a few independent sampling stations per field site. In the *Posidonia* National Monitoring Protocol for Croatia, for example, Guala et al. [19] call for only three such stations per site at the lower seagrass margin. At a sample size of *n* = 3, this protocol is a blind sentinel to losses less than roughly 50% whether these are fixed or random plots (Figs 1 and 2). Even if every single point in a sampled meadow declines by exactly 10% between two monitoring events, nevertheless the chance that the mean of a sample of *n* = 3 independent data points shows a significant decline will be barely over *α* = 0.05. A statistical power of 50% is equivalent to a coin toss; a statistical power of 5% is blind to a true decline 95% of the time. This is just one example of many in the seagrass monitoring literature; approximately a third of the *Posidonia* monitoring methods reviewed by [29] use random-plot direct sampling designs, which is a venerable traditional method [10].

In addition to low statistical power caused by natural variability, random-plot direct methods also suffer from a variety of sampling issues and challenges that are difficult or impossible to address in a routine monitoring program [29]. Most fundamentally, statistical analysis of monitoring data requires that sample points are obtained at random from the same fixed sampling region each monitoring event. This assumption can be violated in many ways when sampling e.g. seagrass shoot density. If the substrate is variable, then the sampling protocol might focus only on substrates capable of supporting seagrass. But if substrate boundaries are gradual, irregular, or difficult to identify, then subjective decisions must be made and are vulnerable to change each monitoring event. If shoot density is sampled only in patches containing seagrass, and if seagrass patches “move” between monitoring events because of progression and regression dynamics, then this sampling protocol cannot see changes in mean shoot density caused by regression and progression, and any change is meaningless in the sense that it cannot be ruled out as an artifact of a change in the region sampled. If, for example, half the bed regresses in regions of low shoot density, then the mean shoot density of the previous bed has plummeted, but if only present patches are sampled, the measured mean shoot density might remain constant or increase. If workers subjectively assess shoot density (or closely related habitat variables) in order to choose where to sample shoot density, then any differences between two monitoring events cannot be distinguished from differences in subjective decisions regarding the locations of sampling points.

Second, depth of the seafloor is a necessary covariate in analyses of shoot density, but if depth is measured by personal SCUBA depth gauges with limited accuracy, then the actual depths within a depth sampling stratum will differ between monitoring events, and therefore create an additional sampling bias that obscures any real difference. *Posidonia* shoot density can decline by about 5–10% per meter of depth [70], and SCUBA depth gauges have an accuracy and precision close to 1.5 meter. Therefore a difference of 10% may be caused simply by a different depth gauge used at different monitoring events. These issues are critical when statistical analysis must rely on just two monitoring endpoints, because limited funding and monitoring urgency do not allow the luxury of a long-term time series. Thus, random-plot direct methods, especially under challenging field conditions presented by subtidal seagrasses, are likely to be distorted by a variety of biases and sampling irregularities whose magnitude is unknown and probably unknowable, in addition to the very low statistical power even under the best sampling design.

For all these reasons, we are in full agreement with the recent recommendation of [57] (p. 147) that, to avoid “simplistic mistakes”, or rampant Type I and II errors, shoot density in random-plot methods for *Posidonia* monitoring “should not be routinely used by administrations responsible for the coastal environment.” Shoot density and other descriptors accessible only by SCUBA can be valuable and informative, however, within a direct, fixed-plot sampling method.

### 3.2 Fixed-plot methods for monitoring cover or shoot density have very high statistical power

Statistical power can be increased by either reducing the variance of the method, or increasing the number of independent samples (Eqs 4 and 5). The obvious way to reduce the error of a random-plot sampling design is to convert it to a fixed-plot design, in which loss is measured directly at permanent field stations, as exemplified by the balisage method of the *Posidonia* Monitoring Network [56], or the SeagrassNet protocol [59]. The advantage of a fixed-plot design is that the standing spatial variation (in cover or shoot density) has zero effect on power, and the residual variance is just the variance in the difference within fixed monitoring plots between monitoring events [4]. Thus, reconsidering the thought experiment above, if every point in a seagrass meadow experiences a 10% loss between monitoring events, then a fixed-plot method will show the same 10% loss in all plots, and the residual error in the statistical test will be zero and the statistical power 100%, regardless of the sample size, even in a meadow whose standing variation in cover or shoot density is the largest that it can possibly be. Fixed-plot designs also are free from the sampling issues of random-plot designs, because plots are fixed, marked, and completely unambiguous.

Fixed-plot monitoring designs are especially favored in situations where the causal agent for loss is diffuse, as is expected in an aquatic environment where loss is caused by decline in water quality. Fixed-plot designs are also favored in species whose maximum positive change is small, resulting in a low theoretical variance among the differences within the plots [4]. Slow growing seagrasses such as *Posidonia oceanica* are thus especially suited to fixed-plot monitoring designs. Our results show that a fixed-plot design for *Posidonia* monitoring requires just 20 fixed stations to detect a mean meadow regression of a few decimeters if the causal agent is diffuse, or a mean regression of less than one meter if the agent is patchy within the sampling site. In the case of a diffuse agent, this represents approximately 1/100th of the sampling effort of a random-plot design for the detection of 1/10th the rate of *Posidonia* decline (Fig 2).

The second means of increasing the power of a random-plot design is to increase the number of independent random samples of the study site. While a minimum sample size of 2000 per sampling stratum per monitoring event is not feasible for routine monitoring by SCUBA, it is feasible by remote methods of seagrass monitoring.

### 3.3 Ease and high accuracy of RUV yields high power

The remote method that most closely duplicates direct observations of SCUBA is remote underwater videography (RUV), which is assumed to have near-zero classification error in distinguishing live seagrass from surface matte, algae, or unvegetated surface. But RUV is free of the cost, fatigue, and time limitations of SCUBA, and therefore the sampling intensity can exceed 100 times that of SCUBA per field day, resulting in a minimum detectable difference in seagrass cover of about one-tenth that of SCUBA within a random-plots method (Eq 4). RUV is also free of the subjective sampling biases of a direct method, since transects are placed blind to the seafloor, and all data are transparent and can be analyzed independently by any number of workers. RUV can also be used within a fixed-plots sampling design, in which transects are revisited with real-time, submeter-accuracy DGPS, no permanent markers are necessary, and a paired analysis is used [49, 50, 53]. In this case, power is increased to the extent the previous transects can be closely revisited, with power never less than that for the random-plots design [49]. If precise revisitation is possible, then monitoring produces 2D or 3D photomosaics for estimate of cover difference within revisited transects, with statistical power given by Fig 2. The use of paired photomosaics for high-resolution monitoring of coral reef habitat is now common, and there is no reason that this method could not also be used to advantage in seagrass monitoring, especially in the clear waters of the Mediterranean [53, 54, 76].

Remote images may not be sufficient to distinguish among morphologically similar species within some seagrass genera, requiring analysis at the generic level in those rare cases where species do not segregate by depth or other habitat characteristics. Neither SCUBA passive observation nor RUV can identify buried matte; only sediment core samples can demonstrate and quantify buried matte [77]. Dead matte, however, is a natural component of dynamic *Posidonia* beds and is often wrongly interpreted as an indicator of human disturbance [59].

RUV has been used to map and monitor biological resources on the seabed since the 1950s [78], and this method has become standard and widespread since the advent of high-accuracy DGPS. Geospatial RUV is an official monitoring protocol for the seagrass *Zostera marina* in Washington State, USA [79], is the basis of the Shallow Water Positioning System for monitoring seagrass and associated habitat in Biscayne Bay, Florida, USA [53], is an official method of monitoring seagrass and other benthic habitats in Victorian (Australia) Marine National Parks and Marine Sanctuaries [80], has been used for the reporting requirement of the EU Habitats Directive for *Posidonia* monitoring in Italy [44], is a standard method used in Australia for surveillance of all seabed habitats to depths of 50 m (see [81, 82] and references therein), and is a standard method for ground truthing (training and validating) remote maps of the seabed worldwide, e.g. [83–86].

### 3.4 Habitat mapping is neither necessary nor sufficient for habitat monitoring

While RUV can satisfy the 10% monitoring criterion, it gathers imagery of the sea bottom at a rate several orders of magnitude lower than that of acoustic methods such as sidescan or multibeam sonar. These latter methods, as well as aerial photography or satellite imaging, are widely perceived to be superior to RUV and direct methods because they can capture large regions of the sea bottom with a minimum of field effort, and thereby produce high-resolution maps that can serve two functions at one cost: 1) they provide “baselines” of the study region that can be used to compare to all future maps to quantify changes in habitat cover, and 2) they provide a benthic habitat layer in a geographic information system (GIS) that can be used for spatial planning decisions, e.g. [87, 88]. The idea that mapping is a necessary first step in monitoring is nearly ubiquitous in the seagrass literature; indeed it is rare for mapping and monitoring *not* to be treated as if they are the same activity. RAMBOLL [14], for example, treat “mapping” and “monitoring” as equivalent methods of regular surveillance under the EU Habitats Directive. Dekker et al. [89] follow many others in stating on p. 415, “The first step is to provide baseline maps that document the current extent, diversity and condition of the seagrasses. The next step is to establish monitoring programs designed to detect disturbance at an early stage ‥‥ ” and the “monitoring programs” referred to here are aerial maps.

Our results indicate that this perception is incorrect. Although a habitat map of the seabed is necessary for informed spatial planning of any activities that impinge on those habitats (such as regulation of bottom trawling near *Posidonia* meadows, [90]), it is neither necessary nor sufficient for sentinel monitoring of those habitats. First, that baseline maps are not necessary as a precondition for monitoring is illustrated by 1) the ongoing annual RUV monitoring of *Zostera marina* along 1000 km of the Washington State USA coastline since the year 2000, in the absence of a habitat map of the benthos or seagrass baseline other than the initial RUV transects themselves [79], 2) the global success of the SeagrassNet monitoring program, started in 2001 in the absence of any global collection of benthic maps [91], 3) the success of the *Posidonia* Monitoring Network begun in France in 1984 in the absence of any baseline maps of the region monitored [56], and 4) the many published BACI studies conducted without any prior map of the sampling areas, e.g. [92]. The ecological literature is replete with examples of statistical sampling and analysis methods for estimating ground cover and its change, without the use of “maps;” see [4] and references therein. Certainly cartography will always be useful to the extent that it is accurate, and will assist the planning of sampling locations [93], but there is no evidence that it must precede any successful monitoring program, and managers need not wait for the creation of maps prior to initiating and funding urgent sentinel monitoring.

Second, a map with unknown classification error is not sufficient by itself for monitoring. Such a map has scientific value only if its accuracy and precision are known and reported, along with the standard errors of all estimates of habitat cover and its change [33, 94]. If these are not reported, then the scientific value of habitat coverage estimated from the map is simply not known. In order for a comparison from two maps to satisfy the 10% monitoring criterion, the classification error of each must be well below the utility threshold of 0.15, a formidable goal for any variable substrate in the marine environment. However, demonstrating that this condition is met introduces a Catch-22 situation: we first must quantify the classification error using a ground truthing study that compares the map classifications to the real habitat on the ground. But if we find that the classification error is not near zero in one of the maps, then the data from the ground truthing study will provide a far more precise “baseline” and estimate of habitat loss than the corrected map, especially if that ground truthing study used a fixed-plots design with balisage or RUV. This brings us back to square one, where the map is not sufficient to satisfy the 10% criterion in the absence of more field effort than would be required by a direct estimate of habitat loss. Thus, in retrospect, a well-designed monitoring study would have been a better investment for monitoring than the production of a habitat map.

This Catch-22 is well illustrated by the case of *Posidonia*. Acoustic or remote sensing maps are generally not accurate enough to satisfy the 10% criterion for *Posidonia*, for two reasons: first, classification error biases estimates of habitat coverage. This is simply an algebraic fact (Eq 11). Classification errors for mosaics of seagrass and other structured habitat (boulders, gravel, shells, macroalgae) are rarely less than 0.3 in acoustic maps (e.g. overall error 0.28 for sidescan sonar *Posidonia* versus consolidated in [95]; 0.30 for single-beam sonar seagrass versus oyster shells in [96]; close to 0.5 in single-beam sonar *Posidonia* versus macroalgae in [34]; close to 0.5 for multibeam sonar backscatter *Posidonia* versus gravelly sand in [38]). In contrast, classification errors for seagrass can be lower than 0.1 in the ideal situation of a homogeneous sand substrate with no other vegetation present [37, 96, 97], but this situation is rare for *Posidonia*, which grows on a variety of consolidated and unconsolidated sediment mixtures, and often associated with other algae and seagrass (*Cystoseira*, *Cymodocea*, and in some areas *Caulerpa*; [98]). Again, the in situ fieldwork necessary to establish that these conditions are met could alternatively be used to estimate habitat loss directly. High-resolution, aerial or satellite photography of *Posidonia* can be highly accurate if other vegetation is rare, but at present only at depths less than about 4 m [34, 43, 99], which prevents sentinel monitoring of the critical lower depth margin of *Posidonia* near 30 to 40 m with these methods. Note that all these examples use modern technology that would score high on the map reliability index proposed by [100]. If classification errors for *Posidonia* are generally near 0.3 under realistic field conditions, then even if they remain constant, any changes in *Posidonia* habitat between monitoring events will be discounted by about 60%, due to classification error alone (Eq 11). A 10% loss will generally be perceived as a 4% loss, which if not corrected will almost always be judged non-significant (Fig 3).

Second, classification error is not constant, but varies among monitoring events, which creates additional random noise in estimates of habitat loss. The clearest evidence of this variation is shown in [36], in which *Posidonia* was mapped at 16 sites twice using sidescan sonar, in 1990 and 1991. The discordance between map pairs was high; never less than 30%, and more than 90% at half the locations. If two maps of the same ground are different at 90% of their habitat classifications, then the difference in classification error between the two must be enormous, at least 0.3 at most locations, completely off the grid in Fig 3. Certainly methods of sidescan sonar and global positioning systems have improved since 1991, but it seems unlikely that the standard deviation in classification error has been reduced to less than 0.1 under realistic field conditions. If so, then our results indicate that diachronic cartography of *Posidonia* with sidescan sonar is nearly always blind to habitat losses of about 20% or less (Fig 3), unless cover estimates are corrected with extensive ground truth studies using Eq 14, although we are unaware of a single study that has employed such correction. Similar results likely hold for single-beam and multibeam methods. However, if we take a classification error of 0.3 as a general result for *Posidonia* under realistic field conditions (see above), then the number of ground truth points necessary to correct the map estimate of habitat loss is five to fifty times the number of points that would be needed to estimate the habitat loss directly (Figs 6 and 7). To illustrate, [95] found a *Posidonia* classification error of 0.28 in an innovative vertical sidescan map, constructed with the assistance of ten SCUBA transects, each 400 meters long. Certainly such a map may have many values other than seagrass monitoring. But for the purposes of monitoring, this transect effort alone would reliably detect a 10% loss (Fig 1) [44, 49], and if transects were permanently marked, they would very likely detect a 1% loss with a fraction of the effort required to produce the validated map (Figs 2 and 6).

These considerations indicate that, contrary to assumptions common in the monitoring literature, mapping and monitoring are fundamentally different activities. At the core of the mapping process is the creation of a model to infer the identity of points that are not directly observed. These model points do not have the same value as statistical replicates that real data points do. Monitoring, in contrast, is the use of a probabilistic sampling design to gather a minimum number of real data points to statistically detect a mean temporal difference. Sampling beyond this minimum provides little additional information about mean loss, yet can increase the resolution of a map without limit, while never eliminating map inaccuracy because it is impossible to directly observe more than a tiny fraction of all mapped points on the seafloor. Monitoring and mapping thus have different goals, methods, technical requirements and limitations, information value, probably different labor pools with different expertise, but compete most likely for the same management dollars. Managers considering cartography as a method of government-mandated monitoring should carefully consider these fundamental differences before investing resources in a map that will always have questionable accuracy, if a fraction of those resources could alternatively be invested in a very precise and powerful sentinel monitoring design.

### 3.5 Extra-statistical considerations

Given that both fixed-plot direct methods and RUV satisfy the 10% criterion, on what further basis should managers decide between these two approaches? The advantage of the fixed-plot direct method is the ability to measure several descriptors, including shoot density, morphology, growth, and productivity at the fixed plots [55, 101]. The disadvantage is that permanent underwater markers are required, along with regular replacement and maintenance [55]. Permanent markers, especially massive ones such as concrete blocks, eliminate the habitat in their footprint, alter strength and direction of currents, sedimentation patterns, and perhaps the suitability of neighboring seagrass habitat. These considerations may or may not be pertinent for managers, and may be site-dependent; e.g., a pristine protected area versus a marina development.

While RUV is limited in the descriptors that can be measured and requires office labor, expertise in photogrammetry, and clear water with at least two meters visibility, it provides 1) a precise measure of habitat cover and its statistical properties including spatial autocorrelation patterns; 2) quantitative measures of density of other species (e.g. *Pinna nobilis* associated with *Posidonia*, other recognizable invertebrates and epiphytic cover); 3) quantification of substrate identity, including exposed matte; and 4) a permanent georeferenced photographic archive for future research. Furthermore, it is the only method currently known that is capable of satisfying the 10% criterion throughout the depth range of seagrasses without the need for direct field (SCUBA) observation or permanent ground markers.

This paper is not intended as an exhaustive treatment of all possible seagrass monitoring methods, and it is certainly possible that others may be demonstrated to satisfy the 10% criterion. For example, it seems almost certain that acoustic telemetry provides power similar to balisage [102], but at present there are no studies of propagation of its measurement error to habitat cover estimates. There may be descriptors other than cover or shoot density that are spatially uniform enough to be used in a direct, random-plot method, e.g. morphological, demographic, or chemical. But regardless of their prevalence, spatial cover will usually be necessary in addition, because cover is a fundamental economic value, and is the only legally required descriptor for habitat monitoring under the EC Habitats Directive. Thus it is likely that any seagrass monitoring program will need to accommodate cover and the statistical implications of its high spatial variability discussed here, regardless of additional descriptors used.

### 3.6 Conclusions

Blind sentinel methods, i.e. generally incapable of detecting 10% habitat losses at a statistical power > 0.5, include direct random-plots methods of monitoring subtidal, patchy seagrass cover and shoot density, and remote mapping methods with non-trivial levels of classification error, such as sidescan, multibeam, and single-beam sonar. Aerial or satellite imagery may provide methods that satisfy the utility threshold for reliably detecting 10% loss in most seagrasses, but these methods currently are useful for sentinel monitoring only at depths shallower than about four meters. Acoustic methods can detect homogeneous seagrass on homogeneous sand with near 100% accuracy, but these field conditions are rare in *Posidonia* and where other vegetation or hard structure is mixed with seagrass. Resource management and regulatory agencies should recognize that mapping and monitoring are two independent activities with conflicting goals and methods, and that remote *Posidonia* maps are generally not capable of dependably detecting less than 30–50% seagrass loss. We found two classes of methods powerful enough to reliably detect a 10% loss of seagrass habitat throughout the natural depth range: direct, fixed-plot methods, and remote underwater videography (RUV), the only remote method with near zero classification error. These should be considered the gold standards for seagrass sentinel monitoring across all substrates and depths. The former include the balisage method used in the *Posidonia* Monitoring Network, and the SeagrassNet global seagrass monitoring method. The only method we found capable of satisfying the 10% criterion throughout the depth range of seagrasses, without the need for SCUBA or direct observation, and without any habitat alteration, is RUV. Non-destructive methods that can reliably detect 10% loss in seagrasses do exist, and can be relied on to prevent further declines in all species. For these methods to become international standards, however, management and regulatory powers must recognize that rigorous and reliable science is the cornerstone of all management success, and formalize this idea with explicit requirements for minimum precision, power, and detectable losses in the official protocols that create the sentinels that watch over these valuable resources.
